# Linguistic validation of the Spanish version of the Anal Cancer High-Grade squamous intraepithelial lesions outcomes Research Health-Related Symptom Index (A-HRSI): AMC-A04

**DOI:** 10.1186/s41687-022-00515-1

**Published:** 2022-10-11

**Authors:** Thomas M. Atkinson, Kathleen A. Lynch, Jacqueline Vera, Nuria Mendoza Olivares, Andrew Webb, Lisa C. Diamond, Javier González, Erica I. Lubetkin, Gary Bucher, Isabella Rosa-Cunha, J. Michael Berry-Lawhorn, Rebecca Levine, David Aboulafia, Jeffrey Schouten, Susan M. Holland, David Cella, Joel M. Palefsky

**Affiliations:** 1 Department of Psychiatry & Behavioral Sciences, Memorial Sloan Kettering Cancer Center, 641 Lexington Ave., 7th Floor, 10022 New York, NY USA; 2 New York University, New York, NY USA; 3 CUNY School of Medicine, New York, NY USA; 4 Anal Dysplasia Clinic MidWest, Chicago, IL USA; 5 Jackson Memorial Hospital, Miami, FL USA; 6 University of California-San Francisco, San Francisco, CA USA; 7 Montefiore Medical Center, New York, NY USA; 8 Virginia Mason Medical Center, Seattle, WA USA; 9 Northwestern University Feinberg School of Medicine, Chicago, IL USA

**Keywords:** Neoplasms, Patient reported outcome measures, Quality of life, Patient outcome assessment, Anal cancer

## Abstract

**Objectives:**

The Anal Cancer High-grade squamous intraepithelial lesions (HSIL) Outcomes Research (ANCHOR) Health-Related Symptom Index (A-HRSI) is a 25-item measure that assesses physical symptoms and impacts, and psychological symptoms. To promote generalizability and equity in the capture of these concepts in Spanish-speaking participants, we linguistically validated a Spanish version of A-HRSI.

**Methods:**

Following independent forward translation and reconciliation of A-HRSI from English to Spanish, two rounds of cognitive interviews were completed with ANCHOR participants who had been diagnosed with anal HSIL in the prior nine months and preferred delivery of their healthcare in Spanish. Interviews were coded to highlight any items and concepts that were reported as being difficult for any reason by ≥ 3 participants, with such items revised during a research team panel discussion and tested in a second round of interviews if applicable.

**Results:**

Seventeen participants representing 8 nationalities were enrolled (Round 1 *n*=10, Round 2 *n*=7); 7 participants reported not completing high school (41.2%). No difficulties were reported with respect to the theoretical concepts measured by A-HRSI. We made modifications to the Spanish translation of eight items and two response option terms in cases where participants had difficulty understanding a term, experienced problems in discriminating between terms, or preferred the use of an alternative term to represent the concept(s).

**Conclusion:**

The Spanish version of A-HRSI is a linguistically valid tool that can be used to assess physical symptoms, impacts, and psychological symptoms related to anal HSIL.

**Plain English Summary:**

Language is a tremendous barrier to enrolling patients to clinical trials. The anal cancer high-grade squamous intraepithelial lesions (HSIL) outcomes research [ANCHOR] trial is a randomized clinical trial that recently established that the treatment of anal HSIL, versus active monitoring, is effective in reducing incidence of anal cancer in persons living with HIV (PLWH). The ANCHOR Health-Related Symptom Index (A-HRSI) is a 25-item patient-reported outcomes measure that was developed to assess physical symptoms, physical impacts, and psychological symptoms related to anal HSIL. As approximately 10% of ANCHOR participants preferred the delivery of their healthcare in Spanish, the purpose of the present study was to linguistically validate a Spanish version of A-HRSI. Based on feedback from interviews with 17 participants from the ANCHOR trial who had been diagnosed with anal HSIL in the prior nine months and preferred delivery of their healthcare in Spanish, we made modifications to the Spanish translation of eight items and two response option terms in cases where participants had difficulty understanding a term, experienced problems in discriminating between terms, or preferred the use of an alternative term to represent the concept(s). The Spanish version of A-HRSI is a linguistically valid tool that can be used to assess physical symptoms, impacts, and psychological symptoms related to anal HSIL as part of clinical trials or routine care.

## Introduction

Anal cancer is increasing in incidence in the United States (US) (i.e., 1.9/100,000 from 2013 to 2017 [1]). People living with HIV (PLWH) are disproportionally affected and have an approximately 161 times higher incidence of anal cancer [2, 3]. While vaccination against human papillomavirus (HPV) is the primary preventative practice [4, 5], secondary prevention has focused on screening for and treating precancerous HPV-associated anal high-grade squamous intraepithelial lesions (HSIL) to prevent progression to cancer [6, 7]. In 2014 the US National Cancer Institute (NCI) funded the Phase III Anal Cancer/HSIL Outcomes Research (ANCHOR) randomized controlled trial (clinicaltrials.gov identifier: NCT02135419), which determined that treatment of anal HSIL, versus active monitoring, is effective in reducing incidence of anal cancer in PLWH [8].

Patient-reported outcomes (PROs) are considered to be the gold standard for the capture of the status of a patient’s health condition (e.g., health-related quality of life; HRQoL) [9]. ANCHOR presented a unique opportunity to gain an understanding of how treatment or active monitoring for anal HSIL may impact immediate and long-term aspects of HRQoL for enrolled participants, however no PRO tool existed for the capture of HRQoL related to anal disease in PLWH. As such, we developed the ANCHOR Health-Related Symptom Index (A-HRSI), a 25-item measure that assesses participant physical symptoms (9 items), physical impacts (7 items), and psychological symptoms (9 items) over the past 7 days via a numeric rating scale (i.e., 0, not at all; 1, a little bit; 2, somewhat; 3, quite a bit; 4, very much) [10–12]. A major limitation of A-HRSI is that this measure has heretofore only been available in English.

Language has been identified as a barrier to clinical trial accrual [13, 14]. To ensure generalizability and equity in clinical trials, it is essential that all aspects of a trial are inclusive to participants with limited English proficiency (LEP). One method to ensure that PROs are inclusive to LEP participants is linguistic validation, defined as the process of assessing and confirming the conceptual equivalence and content validity of translations of PROs, whereby translated text is actively tested with patients in the population and language of interest via cognitive interviews [15, 16]. Cognitive interviewing is an iterative semi-structured methodology through which cognitive processes (i.e., comprehension, memory retrieval, judgement, and response mapping) that are required to complete a PRO tool are evaluated [17]. As approximately 10% of ANCHOR participants preferred the delivery of their healthcare in Spanish, the purpose of the present study was to linguistically validate a Spanish version of A-HRSI.

## Methods

### Participants

Participants were eligible if they were Spanish speaking with LEP (as assessed by a single item that asked whether the individual prefers to have their healthcare delivered in Spanish [18]) and had been diagnosed with anal HSIL within the prior nine months. All data collection was centrally coordinated through Memorial Sloan Kettering Cancer Center (MSK; New York, NY). This study was reviewed and deemed exempt by the US NCI’s Cancer Therapy Evaluation Program and the institutional review boards at each participating study site.

### Measure translation

A-HRSI was translated from English to Spanish at MSK in accordance with best practices in PRO translation [16]. This included a process of forward translation from English to Spanish by a certified translator, independent review and reconciliation by a second certified translator, and a final review and reconciliation by a third certified translator into a single translation of A-HRSI.

### Procedure

Each of the 17 US ANCHOR sites was polled for study feasibility. Participating sites were asked to distribute a study information sheet to potentially eligible individuals. This sheet included a referral number, coded information on ANCHOR arm assignment and anal disease volume, and encouragement language in Spanish to contact the Clinical Research Coordinator (CRC) at MSK via telephone to confirm eligibility and schedule a time to participate.

Each interview was completed via telephone, where participants were first asked to complete A-HRSI, as aurally administered by the native Spanish speaking interviewer (JV or NMO), indicate whether there were any items or aspects of the instructions that were difficult to understand, and then complete a process of retrospective probing. Participants received a $50 US Postal Service money order for their time and effort. All interviews were audio-recorded and stored on an MSK secure server for a maximum of 48 business hours to facilitate the completion of summary reports.

### Analytic approach

An *a priori* sample of 10 participants were to complete Round 1 (R1) of interviews. Summary reports from each R1 interview were coded by the interviewer and a qualitative research analyst (KAL) to highlight any instances where a participant indicated that they experienced difficulty with an item or concept within an item. For any item or concept that was reported by at least three participants as difficult for any reason, that item or concept was reviewed to determine whether the difficulty was due to (1) the concept being measured or (2) a translation issue (i.e., register, jargon, or regionalism). These items were revised during a research team panel discussion and used in Round 2 (R2) interviews with additional eligible participants. A similar process was used for R2, where we again determined whether item difficulties were due to conceptual- or language-related issues, with final changes to the measure made during a research team panel discussion.

## Results

Five ANCHOR sites (i.e., Jackson Memorial Hospital [Miami, FL], Anal Dysplasia Clinic-MidWest [Chicago, IL], University of California – San Francisco [San Francisco, CA], Virginia Mason Medical Center [Seattle, WA], and Montefiore Medical Center [New York, NY]) agreed to refer eligible participants to the study. The remaining ANCHOR sites either did not enroll Spanish-speaking participants (*n*=9) or did not consider the study to be exempt research (*n*=3). Between November 2018 and January 2020, 51 study information sheets were distributed. Twenty potentially eligible participants contacted the CRC to confirm their eligibility, with 3 participants lost to follow up.

Two rounds (i.e., R1 *n*=10, R2 *n*=7) of cognitive interviews were completed with a total of 17 participants (Table 1; median age = 52 years, 70.6% cisgender male [*n*=12], 17.6% cisgender female [*n*=3], 11.8% transgender female [*n*=2]) referred from 3 sites. All participants identified as Hispanic and 82.4% [*n*=14] identified as white. Eight nationalities were reported across participants (i.e., Cuban [*n*=4], Venezuelan [*n*=3], Puerto Rican [*n*=3], Mexican [*n*=2], Honduran [*n*=2], Nicaraguan [*n*=1], Costa Rican [*n*=1], and Columbian [*n*=1]). Seven participants indicated that they did not complete a high school education (41.2%).

Table 2 includes a summary of terms from the Spanish version of A-HRSI that were modified after R1 or R2, including reasons for modification. There were no difficulties reported with respect to the theoretical concepts being measured, however there were numerous changes made to the English-to-Spanish translations of content that represents these concepts. The completion instructions were understood by all participants; no modifications were required for the translations of this content.

### Response category

During R1, four participants expressed difficulty discriminating between terms for “quite a bit (bastante)” and “very much (mucho)” and viewed these terms as interchangeable. To create a greater distinction between the concepts being measured, “mucho” was changed to “muchísimo,” with no additional difficulties reported for this term in R2. Similarly, during R2, the Spanish translated term that was used for “not at all (para nada)” was characterized by three participants as an “unusual” expression and was seen as difficult to distinguish from the concept of “not applicable.” As such, “para nada” was changed to “nada” after R2.

### Physical symptoms

As part of R1, three participants expressed difficulty in understanding “defecación” in the context of urgency or pain related to bowel movements. Given this feedback, the item “tengo dolor durante la defecación” was changed to “tengo dolor cuando entro al baño a defecar,” and the item “siento urgencia de defecar” was modified as “siento urgencia de entrar al baño a defecar” to enhance understanding and provide additional context. No further changes were required for these items after R2.

### Physical impacts

When responding to “I have problems taking care of myself (e.g., bathing dressing, shaving) (tengo problemas con mis deberes personales diarios (por ej., bañándome, vistiéndome, afeitándome),” three participants indicated that the phrase “deberes personales” was unclear to them and that this did not relate to the daily care activities that were used as examples. To improve understanding, this was changed to “problemas con mi arreglo personal” after R1.

R2 interviews resulted in several changes to the Spanish translations for content related to physical impacts. For the item “I have problems with my physical ability to move around (tengo problemas con mi capacidad física para movilizarme),” three participants stated that “movilizarse” was not specific enough to the act of physical movement and interpreted this term as referring to “problems mobilizing myself” or “motivation.” To potentially remove ambiguity, the term was modified as “tengo problemas con mi capacidad física para moverme.” Two issues were raised by three participants with the item “I have problems completing daily household chores (e.g., cleaning, cooking, laundry, house maintenance) (“tengo problemas terminando las tareas de la casa (por ej., limpiar, cocinar, hacer la colada, administrar la casa);” the term “tareas” was confusing to participants and the example “hacer la colada” was characterized as “too regional” or “unfamiliar.” Participants suggested use of “preparar el café” as an alternative example. The item was changed to “tengo problemas manteniendo la casa (por ej., limpiar, cocinar, preparar el café, administrar la casa).” For the item “I have problems participating in leisure activities (e.g., watching television, relaxing) (tengo problemas participando en actividades de ocio (por ej., mirar televisión, relajarme),” the term “ocio” was viewed by three participants as an “archaic” phrase for “leisure” and was replaced with “relajo.”

### Psychological symptoms

During R2, when asked about “decreased enjoyment of anal sexual activity” or “decreased enjoyment for any form of sexual activity other than anal sexual activity,” these items were interpreted in terms of desire rather than physical pleasure. Given this feedback from three participants, the term “placer” was replaced with “disfrute” (e.g., “me ha disminuido el disfrute de la actividad sexual anal” and “me ha disminuido el disfrute de cualquier forma de actividad sexual diferente a la actividad sexual anal”).

## Discussion

Inclusion of LEP participants in clinical trials is essential to ensure generalizability of research findings [19, 20]. Unfortunately, a recent review of over 14,000 clinical trials in the US from 2019 to 2020 found that 18.98% specifically excluded non-English speakers, with only 2.71% of trials accommodating participant languages other than English [21]. Toward increasing inclusiveness in ANCHOR and clinical trials that include a similar cohort of participants, we developed a linguistically validated version of A-HRSI using an iterative process, including two rounds of cognitive debriefing interviews that helped refine the translation quality.

The current study may potentially be limited by a small sample size (i.e., *N*=17), however a strength of this sample is its diversity and representativeness; eight different nationalities were represented across participants, two participants identified as transgender (11.8%), and seven participants reported not having completed a high school education (41.2%). Additionally, the number of participants is consistent with recommendations in the literature for cognitive interviewing [17, 22]. This study predated the SARS-CoV-2 (COVID-19) pandemic, which saw rapid rise in use and acceptance of virtual technologies to complete qualitative interactions [23–25]. While all interviews in the present study were completed virtually via telephone, utilization of videoconferencing platforms (e.g., Zoom, Skype, Microsoft Teams) should be used as a standard for virtual cognitive interviews when technologically feasible to allow for an interviewer to observe participant behavior (e.g., changes in body language, gestures, facial expressions) as they respond to an item and use follow-up probes to determine whether these nonverbal cues are related to completing the questionnaire.

The Spanish version of A-HRSI is a culturally- and linguistically sensitive questionnaire that we expect will be useful in the Spanish-speaking anal cancer and HSIL patient community beyond the context of ANCHOR. Elimination of language barriers to PRO assessment is an important step to integrating participant voice into clinical decision-making.

**Table 1:** Participant demographics and clinical characteristics

	Total (*N=17*)	(%)
**Age (years)**		
Mean (SD)	50.5 (9.98)	-
Median	52	-
Range	36–68	-
**Gender Identification**		
Cisgender Male	12	(70.6%)
Cisgender Female	3	(17.6%)
Transgender Male	0	(0.0%)
Transgender Female	2	(11.8%)
**Racial Identification**		
White	14	(82.4%)
Other	3	(17.6%)
**Ethnic Identification**		
Hispanic	17	(100.0%)
**Nationality**		
Cuban	4	(23.5%)
Venezuelan	3	(17.6%)
Puerto Rican	3	(17.6%)
Mexican	2	(11.8%)
Honduran	2	(11.8%)
Nicaraguan	1	(5.9%)
Costa Rican	1	(5.9%)
Colombian	1	(5.9%)
**Education**		
< High School	7	(41.2%)
≥ High School	10	(58.8%)
**Referring Site**		
Miami	14	(82.4%)
Anal Dysplasia Clinic MidWest	2	(11.8%)
Montefiore	1	(5.9%)
**Study Arm**		
Active Monitoring	10	(58.8%)
Treatment	7	(41.2%)
**Lesion Volume**		
High Grade (> 50%)	2	(11.8%)
Low Grade (≥ 50%)	15	(88.2%)

**Table 2:** Spanish A-HRSI modified terms and reasons for modifications

Domain	Item in English	Original item in Spanish	Revised item in Spanish	Reason(s) for the modification
**Response Category**				
	Very much- 4	Mucho- 4	Muchísimo- 4	Participants had difficulty discriminating between “bastante” (3) and “mucho” (4); these were overlapping and/or interchangeable terms (R1)
	Not at all- 0	Para Nada- 0	Nada- 0	Para nada seen as an “unusual” expression or difficult to distinguish from “Not applicable” (R2)
**Physical Symptoms**				
	I have pain during bowel movements	Tengo dolor durante la defecación	Tengo dolor cuando entro al baño a defecar	Participants had difficulty understanding the noun “defecación” (defecate/ defecating) (R1)
	I have urgency for bowel movements	Siento urgencia de defecar	Siento urgencia de entrar al baño a defecar	
**Physical Impacts**				
	I have problems taking care of myself (e.g., bathing, dressing, shaving)	Tengo problemas con mis deberes personales diarios (por ej., bañándome, vistiéndome, afeitándome)	Problemas con mi arreglo personal	“Problemas personales” (“problems with everyday duties”) was unclear to participants—they did not interpret this to mean daily care activities such as bathing, brushing teeth, etc. (R1)
	I have problems with my physical ability to move around	Tengo problemas con mi capacidad física para movilizarme	Tengo problemas con mi capacidad física para moverme	“Movilizarse” is not specific enough to the act of physical movement (e.g. walking). Some participants interpreted this to mean “problems mobilizing myself”, or motivation (R2)
	I have problems completing daily household chores (e.g., cleaning, cooking, laundry, house maintenance)	Tengo problemas terminando las tareas de la casa (por ej., limpiar, cocinar, hacer la colada, administrar la casa)	Tengo problemas manteniendo la casa (por ej., limpiar, cocinar, preparar el café, administrar la casa)	“Tareas” was initially confusing to participants; “hacer la colada” was seen as unfamiliar or “too regional”; “preparar el café” is a more widely understood example (R2)
	I have problems participating in leisure activities (e.g., watching television, relaxing)	Tengo problemas participando en actividades de ocio (por ej., mirar televisión, relajarme)	Tengo problemas participando en actividades de relajo (por ej., mirar televisión, relajarme)	“Ocio” viewed as an “archaic” phrase for “leisure”, difficult to understand (R2)
**Psychological Symptoms**				
	I have a decreased enjoyment of anal sexual activity	Me ha disminuido el placer de la actividad sexual anal	Me ha disminuido el disfrute de la actividad sexual anal	Participants often interpreted these items in terms of desire, rather than physical pleasure. Changed to “placer” to “disfrute” to specify physical enjoyment (R2)
	I have a deceased enjoyment for any form of sexual activity other than anal sexual activity	Me ha disminuido el placer de cualquier forma de actividad sexual diferente a la actividad sexual anal	Me ha disminuido el disfrute de cualquier forma de actividad sexual diferente a la actividad sexual anal	

Note: R1 indicates modifications were made after Round 1 of cognitive interviews. R2 indicates modifications were made after Round 2 of cognitive interviews. No modifications were required for the completion instructions.

## Data Availability

The datasets used and/or analyzed during the current study are available from the corresponding author on reasonable request.

## List of abbreviations

A-HRSI: Anal Cancer High-Grade Squamous Intraepithelial Lesions Outcomes Research Health-Related Symptom Index
ANCHOR: Anal Cancer High-Grade Squamous Intraepithelial Lesions Outcomes Research
CRC: Clinical Research Coordinator
HPV: Human Papillomavirus
HRQoL: Health Related Quality of Life
HSIL: High-Grade Squamous Intraepithelial Lesions
LEP: Limited English Proficiency
MSK: Memorial Sloan Kettering Cancer Center
NCI: National Cancer Institute
PLWH: People Living with HIV
PRO: Patient-Reported Outcome
R1: Round 1
R2: Round 2
US: United States
